# Surgical management of organizing pneumonia: a retrospective study of 24 cases in a single Centre

**DOI:** 10.1186/s13019-019-0939-2

**Published:** 2019-06-28

**Authors:** Ge Yu, Huaijun Ji, Chuizheng Meng, Yixuan Huang, Guogang Gao, Chuanping Liu, Shanlei Wang, Lei Zhang, Jin Ju

**Affiliations:** Department of Thoracic Surgery, Weihai Municipal Hospital, 70 Heping Road, Weihai, 264200 Shandong China

**Keywords:** Organizing pneumonia, Surgical management, Corticosteroid treatment

## Abstract

**Background:**

Organizing pneumonia (OP) is a rare disease that is often easily misdiagnosed as a malignancy. The diagnosis of OP can prove quite challenging. Patients typically receive treatment with high-dose corticosteroids. Relapse is common if corticosteroid treatment is reduced or stopped. However, given that long-term corticosteroid treatment often results in significant side-effects, the aim of this study was to discuss the diagnosis and surgical treatment of OP.

**Material and methods:**

The medical records of 24 patients with pathologically diagnosed OP between October 2007 and January 2019 were retrospectively reviewed. All patients underwent thoracic computed tomography (CT) and transbronchial biopsy or CT-guided percutaneous needle aspiration. We analysed the clinical manifestations, radiological findings, diagnostic methods, treatment, and follow-up outcomes of all patients.

**Results:**

In total, 24 patients with OP were identified. The study included 17 (70.8%) men and 7 (29.2%) women, and the mean age was 61.25 ± 11.33 years (range: 31–82). The most common symptom was cough (*n* = 16; 66.6%), and the most common radiological finding was consolidation (*n* = 13; 54.2%) on thoracic CT. The diagnosis of OP was made by transbronchial biopsy in 11 patients (45.8%), and percutaneous needle aspiration biopsy in 13 (54.2%). We performed 11 wedge resections, 9 segmentectomy, and 4 lobectomies. Twenty patients underwent video-assisted thoracoscopic surgery (VATS), and 4 underwent thoracotomy. Complete lesion resection was obtained in all patients, and all patients were discharged from the hospital between 5 and 11 days after surgery. The mean follow-up period was 59.1 ± 34.5 (range: 2–134) months. Residual lesions or local or distant recurrence were not observed.

**Conclusions:**

OP is a rare disease, and the exact aetiology remains unclear. Preoperative diagnosis is difficult to achieve despite the use of transbronchial biopsy or CT-guided percutaneous needle aspiration. Complete surgical resection represents an effective method for the treatment of OP.

## Introduction

Organizing pneumonia (OP) is a common histopathological response to injury in the lung, demonstrating inflammatory intra-alveolar infiltration that leads to fibrosis on pathology [1]. OP can be primary or secondary to some clinical situations. When OP occurs as a primary entity with an unknown aetiological cause, it is called “cryptogenic OP (COP)”. Secondary OP (SOP) is associated with various diseases that are known to induce OP, including inflammatory bowel diseases, connective tissue diseases, malignancy, infection, drug reactions, bone marrow or organ transplantation, chemotherapy or radiation therapy for lung or breast cancer, and aspiration [2, 3].

The histopathological pattern of OP typically reveals granulation tissue plugs within the alveoli, alveolar ducts, and small airways (Masson bodies) [2, 4]. These inflammatory tissue plugs occlude the distal bronchioles and previously described bronchiolitis obliterans OP (BOOP) [5]. Differential diagnoses include lung cancer, acute and chronic eosinophilic pneumonia, pulmonary lymphoma and pulmonary vasculitis [6, 7]. Corticosteroids represent the main treatment for OP, but relapses are common after reducing or stopping treatment [8–10]. In this retrospective study, we aimed to report the experience on the surgical management of OP patients.

## Materials and methods

The medical records of 24 patients diagnosed with OP from October, 2007 to January, 2019 in our hospital were reviewed. All patients had no history of malignancy, and haven’t undergone chemotherapy or radiotherapy. All patients were subject to thoracic computed tomography (CT) scans. Diagnosis of OP was made by transbronchial biopsy or CT-guided percutaneous needle aspiration. The demographic data, symptoms, thorax CT findings, diagnostic methods, and treatments were recorded in all cases. All patients were followed between November 2007 and April 2019. This retrospective study was approved by the Institutional Review Board (IRB) of the Weihai Municipal Hospital, and permission was obtained from all participants.

### Statistical analysis

Only descriptive statistics are provided. In descriptive statistics, frequency and percentages were used for categorical variables, and mean ± standard deviation values were used for continuous variables. All recorded data statistical analyses were performed using SPSS 20.0 software.

## Results

Between 2007 and 2019, we retrospectively evaluated 24 patients (17 men and 7 women) with a diagnosis of OP. The mean age of the patients was 61.25 ± 11.33 years (range: 31–82). The most common symptom was cough, sputum, fever, shortness of breath and chest pain (Table 1).

**Table 1:** General characteristics, clinical symptoms, blood tests, pulmonary function test and thoracic CT findings of the patients

Variables	Results
General Characteristics	
Number of patients	24
Age, years	61.25 ± 11.33 (range: 31–82)
Sex (male)	17 (70.8%)
Non-smoker or Ex-smoker	18 (75.0%)
Clinical Symptoms	
Cough	16 (66.6%)
Sputum	10 (41.7%)
Fever	8 (33.3%)
Shortness of breath	6 (25.0%)
Chest pain	5 (20.8%)
Hemoptysis	3 (12.5%)
Weakness	2 (8.3%)
Asymptomatic	4 (16.7%)
Blood Tests	
Elevated CRP	8 (33.3%)
Elevated ESR	6 (25%)
Neutrophilic leukocytosis	5 (20.8%)
Normal	15 (62.5%)
Pulmonary Function Test	
Restrictive pattern	2 (8.3%)
Obstructive pattern	1 (4.2%)
Normal	20 (83.3%)
Radiological Findings (%)	
Consolidation	12 (50%)
Mass-like lesion	6 (25%)
GGO	5 (20.8%)
Spiculated margin	10 (41.7%)
Air bronchogram	7 (29.2%)
Obvious strengthening	6 (25%)
Lymphadenectasis	5 (20.8%)
Pleural effusion	3 (12.5%)
Pleural indentation	2 (8.3%)

Data are presented as n (%). *CRP* C-reactive protein, *ESR* Erythrocyte Sedimentation Rate

**Fig. 1:**
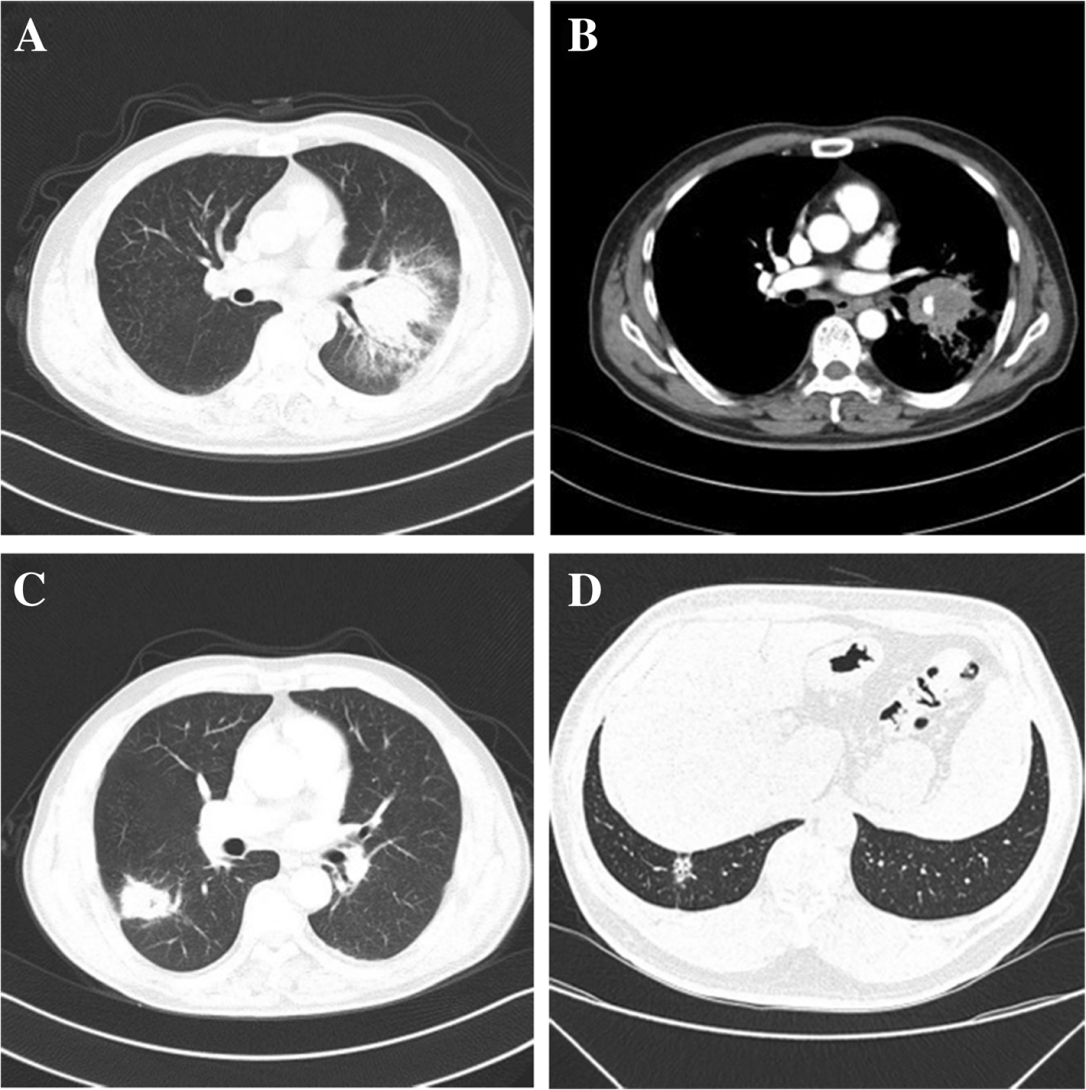
Thorax CT manifestations of OP cases. **a**, **b** OP presenting as a bulky consolidated mass with coarse margins and moderate enhancement in left upper lobe with hilum and mediastinal lymphadenectasis. **c** OP presenting as a mass-like lesion with coarse margins and pleural indentation, and low-density air is noted in the central in the right lower lobe. **d** OP presenting as a predominantly ground glass opacity, air bronchogram and pleural indentation in the right lower lobe

Blood tests were normal in 15(62.5%) patients. Increased erythrocyte sedimentation rate (ESR) values were noted in 6(25%) patients. Increased C-reactive protein values (CRP) were noted in 8(33.3%) patients, and neutrophilic leucocytosis was noted in 5(20.8%) patients. Pulmonary function test (PFT) was normal in 20 (83.3%) of our patients. A restrictive pattern was observed in 2(8.3%) patients, and an obstructive pattern was determined in 1(4.2%) patients. The main radiological manifestation was a consolidated, mass-like lesion and ground glass opacity (GGO) (Fig. 1; Table 1). Three lesions were located in the right superior lobar, 2 in the right middle lobe, 7 in the right lower lobe, 4 in the left upper lobe, and 8 in the left lower lobe. The most common lesion location was the bilateral lower lobe (62.5%). CT scans revealed spicules of margin in 10 (41.7%) patients, air bronchogram in 7 (29.2%) patients, significant enhancement in 6 (25%) patients, lymphadenectasis in 5 (20.8%) patients, pleural effusion in 3(12.5%) patients, and pleural indentation in 2(8.3%) patients. Distant lesions were excluded in all cases.

**Fig. 2:**
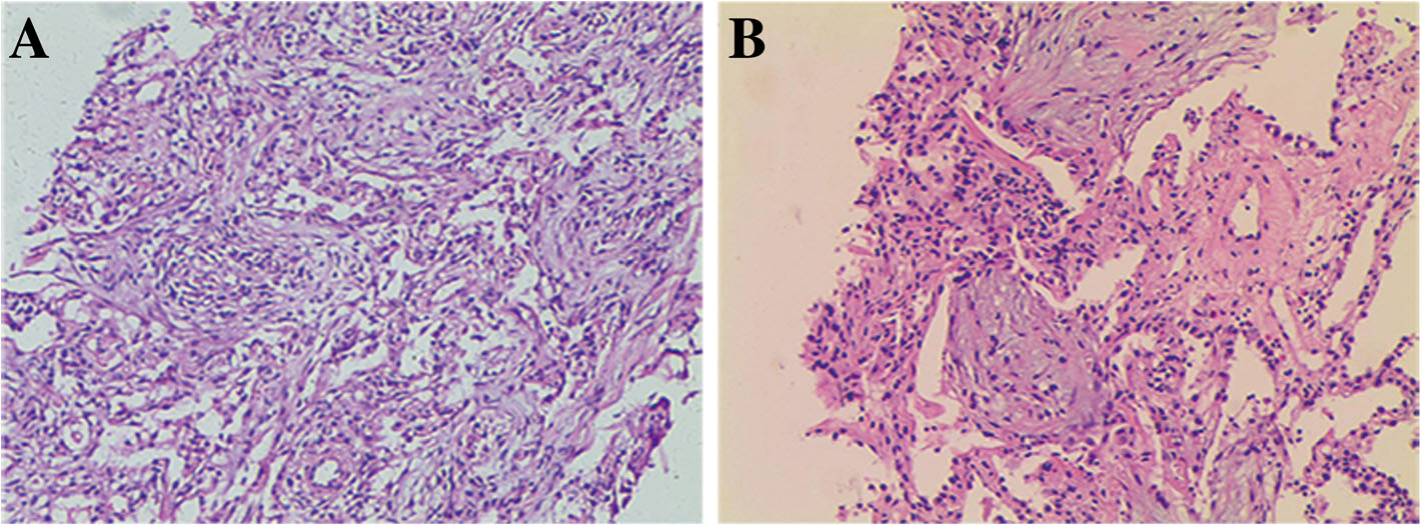
Histopathological features of OP. **a**: The structure of lung tissue was destroyed and the alveolar wall tissue was widened. **b**: The exudation of fibrin in the alveolar cavity was replaced by granulation tissue. (A: 10× H&E; B: 40× H&E)

Transbronchial biopsy was performed in 19 patients, and 14 patients underwent CT-guided percutaneous needle aspiration. Among those, 8 patients experienced one puncture, 2 patients experienced two punctures, 5 patients underwent bronchoscopy once, 4 patients underwent puncture once and bronchoscopy once, 4 patients underwent puncture once and bronchoscopy twice, and 1 patient experienced two punctures and one bronchoscope procedure. The diagnosis of OP was made by transbronchial biopsy in 11 (45.8%) patients, percutaneous needle aspiration in 13 (54.2%) (Fig. 2).

Three patients were directly treated by surgery due to the family history of lung cancer and malignancy cannot be excluded combined with image data, 7 patients were treated by surgery because they had not responded to treatments for 3–6 months, and 14 cannot tolerate the side effect of steroid. We performed 11 wedge resections, 9 segmentectomy, and 4 lobectomies. Among these procedures, VATS was utilized in 20 cases, and open surgery was utilized in 4 cases. The operation time was 91.36 ± 43.60 (35–210) minutes, the blood loss was 83.50 ± 69.45 (20–400) ml, the postoperative hospitalization time was 3.67 ± 1.13 (3-7) days, and the thoracic tube extubation time was 2.50 ± 0.92 (2-5) days (Table 2). Completed lesion resection was obtained in all patients. The postoperative course was uneventful, and patients were discharged from the hospital between 3 and 7 days after surgery.

**Table 2:** Surgical data of the patients

Variables	Results
scope of surgery	
wedge resection	11 (45.8%)
segmentectomy	9 (37.5%)
lobectomies	4 (16.7%)
way of surgery	
VATS	20 (83.3%)
open surgery	4 (16.7%)
General Characteristics	
operation time (min)	91.36 ± 43.60 (35–210)
blood loss (ml)	83.50 ± 69.45 (20–400)
postoperative hospitalization time (d)	3.67 ± 1.13 (3–7)
thoracic tube extubation time (d)	2.50 ± 0.92 (2–5)

*VATS* video-assisted thoracoscopic surgery

The mean follow-up period of the patients was 59.1 ± 34.5 months (range 2 to 134 months). There were no obvious discomfort and complication after discharge. One patient was lost to follow-up. Patients are currently still being followed.

## Discussion

OP is rare. The exact aetiology of this inflammatory reaction remains unclear [11]. OP does not exhibit a sex predilection and is more common in older people, with a mean age of onset of 50–60 years [12]. Rare cases have been reported in children. OP is clearly not related to smoking [10]. Lazor et al. reported that OP is more prevalent in non-smokers or ex-smokers, and the proportion was higher among females [13]. In our study, the mean age of patients was 61.25 ± 11.33 (31–82) years, 17 (70.8%) patients were male, and 18 (75.0%) patients were non-smokers or ex-smokers.

Patients may remain asymptomatic in 30 to 70% of cases [14]. Common symptoms included cough, dyspnoea, fever, weakness, pleuritic chest pain, weight loss and haemoptysis [15, 16]. OP is characterized by a mild flu-like illness with nonspecific symptoms [3]. A diagnosis of OP should be considered in patients with a suspected infective pulmonary disorder not responding to antibiotic therapy [17]. In our patients, the most common symptoms included cough, sputum, fever, shortness of breath, and chest pain, haemoptysis, and weakness. Blood tests and pulmonary function test (PFT) do not make a significant contribution to the diagnosis of OP. In the present study, normal blood test in 15(62.5%) patients, and normal PFT in 20 (83.3%) patients.

There are no specific radiological signs for OP. The most typical imaging pattern of OP is a migratory bilateral patchy alveolar infiltrate [14, 18, 19]. Niksarlıoğlu et al. reported that consolidation was observed in 76.6% of their cases [12]. Maimon et al. found that consolidation was present in 77% of their cases, 86% had ground glass opacities (GGO), and 32% had nodules [20]. Similar to the previous studies, consolidation was observed in 12 (50%) and mass-like lesions were observed in 6 (25%) of our cases.

Spontaneous remission of OP is rare, therefore, the diagnosis of OP typically requires treatment if the patient is symptomatic [21, 22]. OP exhibits rapid clinical, imaging and functional improvements with corticosteroid treatment. Some cases with mild or recurrent OP have good response treated with macrolide [23]. Series of OP associated with rheumatoid arthritis have described good results when treated with methotrexate, cyclophosphamide and azathioprine [24]. Others series show that tocilizumab could be a therapeutic alternative in refractory OP associated with systemic lupus erythematosus (SLE) [23, 25]. Relapses are common upon corticosteroid dose reduction or treatment suspension, thus often leading to prolonged treatment [26–28]. Recurrence rates vary between 9 and 58% [10, 12, 29]. However, long-term corticosteroid treatment often results in significant side-effects, such as gastrointestinal bleeding, bone fracture, diabetes mellitus, arterial hypertension, upper respiratory tract infections, urinary tract infections and body weight increase [30, 31]. In our study, 14 patients were treated by surgery because cannot tolerate the side effect of steroid.

OP is often found in the shadows of lung cancer [32, 33]. The coexistence of OP with lung cancer has been reported [34]. Zhao’s study of 45 patients with lung cancer concluded that OP lesions may resemble lung carcinoma, and OP may be misdiagnosed as a malignancy [1]. Zhao sought to determine CT features that could differentiate OP from lung carcinoma, however, they concluded that small OP lesions may resemble lung carcinoma [18]. In our study, the main radiological manifestation was consolidated, mass-like lesion and GGO. CT scans revealed lesions with irregular margins (41.7%), air bronchogram (29.2%), significant enhancement (25%), lymphadenectasis (20.8%), pleural effusion (12.5%), and pleural indentation (8.3%). These features also represent typical manifestations of lung cancer on imaging modalities. Three patients were direct treated by surgery because suspected lung cancer according to the image. After the development of OP, its pathological changes are irreversible, and loss of normal organizational structure and function is observed. The main component of OP is fibrous tissue. Although the lesion size does not initially increase for a short period of time, it will not shrink or be absorbed. Previous studies have indicated that it may lead to scarring or cancer [35, 36]. So, we think surgery should be performed if the patient cannot tolerate the side effect of steroid or had not responded to treatments.

Complete lesion resection with preservation of uninvolved pulmonary parenchyma remains the fundamental goal in the surgical treatment of OP. If surgical management confirmed, small-range pneumonectomy should be performed, such as segmentectomy or wedge resection, to preserve as much of the functional lung tissue as possible. Hilar lymphadenectomy cannot be recommended as a routine procedure for the treatment of OP. For larger lesions or the mass is located at the hilum, lobectomy can be performed. The frozen pathological sections should be examined during the operation to avoid extending the resection. In our study, 24 patients were treated by surgery. Three patients were treated by surgery because the family history of lung cancer, 7 had not responded to corticosteroid therapy, and 14 cannot tolerate the side effect of steroid. We performed 11 wedge resections, 9 segmentectomy, and 4 lobectomies. We performed 4 lobectomies because those mass is large and close to the hilum of the lung, and wedge resection is very difficult. Among these procedures, VATS was utilized in 20(83.3%) cases, and the postoperative hospitalization time was 3.67 ± 1.13 [3–7] days. We are following them 59.1 ± 34.5 months (range 2 to 134 months). There were no obvious discomfort and complication. The prognosis was good in all patients after the operation, and no long-term complications were noted.

## Conclusions

In conclusion, the exact aetiology of OP remains unclear. Hormone therapy should be considered after a clear diagnosis of OP. We can consider surgical treatment when the patient cannot tolerate the side effect of steroid or had not responded to treatments. At surgery, small-range pneumonectomy should be performed and to preserve as much of the functional lung tissue as possible. Complete surgical resection is a safe effective method for the treatment of OP.

## Data Availability

Data and materials are available.

## Abbreviations

BOOP: Bronchiolitis obliterans organizing pneumonia
COP: Cryptogenic organizing pneumonia
CRP: C-reactive protein values
CT: Computed tomography
ESR: Erythrocyte sedimentation rate
GGO: Ground glass opacity
IRB: Institutional Review Board
OP: Organizing pneumonia
PFT: Pulmonary function test
SLE: Systemic lupus erythematosus
SOP: Secondary organizing pneumonia
VATS: Video-assisted thoracoscopic surgery
